# Footprint of pancreas infiltrating and circulating immune cells throughout type 1 diabetes development

**DOI:** 10.3389/fendo.2023.1275316

**Published:** 2023-11-10

**Authors:** Ylke Bruggeman, Pieter-Jan Martens, Gabriele Sassi, Marijke Viaene, Clive H. Wasserfall, Chantal Mathieu, Conny Gysemans

**Affiliations:** ^1^ Clinical and Experimental Endocrinology, Department of Chronic Diseases and Metabolism (CHROMETA), KU Leuven, Leuven, Belgium; ^2^ Diabetes Institute, Department of Pathology, Immunology and Laboratory Medicine, University of Florida, Gainesville, FL, United States

**Keywords:** type 1 diabetes, immunodynamics, NOD mouse model, single cell RNA sequencing, pancreas

## Abstract

**Introduction:**

Type 1 diabetes (T1D) is defined by immune cell infiltration of the pancreas, in particular the islets of Langerhans, referred to as insulitis, which is especially prominent during the early disease stages in association with decreased beta cell mass. An in-depth understanding of the dynamics and phenotype of the immune cells infiltrating the pancreas and the accompanying changes in their profiles in peripheral blood during T1D development is critical to generate novel preventive and therapeutic approaches, as well as to find biomarkers for the disease process.

**Methods:**

Using multi-parameter flow cytometry, we explored the dynamic changes of immune cells infiltrating the pancreas and the pancreatic draining lymph nodes (PLN), compared to those in peripheral blood in female and male non-obese diabetic (NOD) mice during T1D progression.

**Results:**

The early stages of T1D development were characterized by an influx of innate dendritic cells and neutrophils in the pancreas. While dendritic cells seemed to move in and out (to the PLN), neutrophils accumulated during the pre-symptomatic phase and reached a maximum at 8 weeks of age, after which their numbers declined. During disease progression, CD4^+^ and CD8^+^ T cells appeared to continuously migrate from the PLN to the pancreas, which coincided with an increase in beta cell autoimmunity and insulitis severity, and a decline in insulin content. At 12 weeks of age, CD4^+^ and especially CD8^+^ T cells in the pancreas showed a dramatic shift from naïve to effector memory phenotype, in contrast to the PLN, where most of these cells remained naïve. A large proportion of pancreas infiltrating CD4^+^ T cells were naïve, indicating that antigenic stimulation was not necessary to traffic and invade the pancreas. Interestingly, a pre-effector-like T cell dominated the peripheral blood. These cells were intermediates between naïve and effector memory cells as identified by single cell RNA sequencing and might be a potential novel therapeutic target.

**Conclusion:**

These time- and tissue-dependent changes in the dynamics and functional states of CD4^+^ and CD8^+^ T cells are essential steps in our understanding of the disease process in NOD mice and need to be considered for the interpretation and design of disease-modifying therapies.

## 1 Introduction

Type 1 diabetes (T1D) results from the immune-mediated destruction of the insulin-producing beta cells, leading to insulin deficiency and ensuing hyperglycemia (stage 3). Disease onset is preceded by a long pre-symptomatic phase with the manifestation of beta cell autoimmunity initially without (stage 1), and eventually with dysglycemia (stage 2) (1). The appearance of islet cell autoantibodies defines the earliest stage of pre-symptomatic diabetes and is indicative of islet immune infiltration, known as insulitis. How and when benign autoimmunity transitions to destructive intra-insulitis is still not completely understood, but it is speculated that circulating naïve-like autoreactive CD8^+^ T cells eventually become activated and extravasate into the pancreas by both beta cell- and immune-related factors (2). Various immune cell types, including neutrophils, dendritic cells (DCs), macrophages, T and B lymphocytes have been shown to infiltrate the pancreas of those developing T1D with different spatiotemporal dynamics (3–7). Despite the characteristic disease stages, there exists substantial heterogeneity amongst individuals developing T1D that defines the inconsistency in rate of progression and disease severity, and may also account for varying rates of success in clinical trials assessing disease-modifying strategies in the prevention and reversal of T1D. A greater appreciation of T1D heterogeneity achieved from a more intricate understanding of the immunopathogenesis at the level of the targeted pancreas would allow for better prognoses for those developing T1D and may additionally allow for the design and development of personalized medicine.

While our knowledge of the circulating immune signatures during T1D progression and during immunotherapies in either stage 2 or 3 is rapidly evolving (8–10), little is known on the equivalent pancreas infiltrating subtypes that contribute to beta cell damage and decay. CD8^+^ T cells are the most abundant lymphocytes in human insulitic islets (3), and both polyclonal and islet specific CD8^+^ T cells, with an effector/memory phenotype, have been shown to be more prevalent in the pancreas of individuals with T1D than in those at-risk or healthy controls (11). Yet, data on deeper CD8^+^ T cell subtyping or activation states that may drive the autoreactive responses during T1D progression are largely absent. Also, in the non-obese diabetic (NOD) mouse model, which shares many genetic and immunological features with the human T1D, T cells dominate the pancreatic lesions (12). In addition, neutrophils have been detected in the pancreas in both murine and human T1D during the early stages of disease development, even before autoantibody positivity (5, 13). Neutrophils were mostly detected in the exocrine pancreas, suggesting that also the non-islet pancreatic tissue may be involved in T1D development. In this regard, CD8^+^ T cells were also detected in the exocrine pancreas of T1D individuals without the presence of prominent insulitis (14).

Although the development of high-end technologies has greatly empowered our knowledge, the scarcity of human sample availability remains a major hurdle for studying the immunopathogenesis of human T1D (6, 7). Consequently, the NOD mouse offers the unique opportunity to investigate the ongoing autoimmune responses locally in the pancreas and its draining lymph nodes and to compare these immune footprints at every disease stage with those in the circulation. Moreover, as in human T1D, not all mice go through the different disease stages with similar kinetics.

Here, we performed an in-depth characterization by multi-parameter flow cytometry of the immune system in paired pancreas, draining lymph nodes, and peripheral blood samples from female and male NOD mice during T1D progression (from 2 weeks of age until disease onset). We further interrogated with Cellular Indexing of Transcriptomes and Epitopes (CITE)-sequencing the transcriptional phenotype of pancreatic CD4^+^ and CD8^+^ naïve and memory T cells compared to those in the circulation.

## 2 Research design and methods

### 2.1 Animals

NOD/ShiLtJ mice and NOD.CB17-Prkdc^scid^/J (NOD/scid) of both genders were purchased from Jackson Laboratory (Bar Habor, ME), kept under semi-barrier conditions, and housed according to protocols approved by the KU Leuven Animal Care and Use Committee (Leuven, Belgium; project number P068/2019). NOD mice were screened three times a week for glycosuria (Diastix; Ascensia Diabetes Care, Machelen, Belgium) and consequently considered diabetic if non-fasting blood glucose values exceeded 200 mg/dL for two consecutive days (AccuCheck, F. Hoffmann-La Roche Ltd., Basel, Switzerland).

### 2.2 Insulitis

Five µM sections from formalin-fixed, paraffin-embedded pancreas were collected 50 µM apart and stained with hematoxylin-eosin (H&E). Samples were scored by two independent investigators with light microscopy at resolution 20× and 40×. The following scoring was applied: 0, no infiltration; 1, peri-insulitis; 2, mild insulitis (lymphocyte infiltration in <50% of the islet); 3, severe insulitis (lymphocyte infiltration in >50% of the islet); and 4, completely destroyed.

### 2.3 Insulin content

Pancreas was homogenized in acidic ethanol (91% ethanol, 9% 1 M H_3_PO_4_), incubated overnight at 4°C and sonicated. Insulin concentration in the supernatant was determined by ELISA (Mercodia, Uppsala, Sweden) and normalized to pancreas weight.

### 2.4 Intraperitoneal glucose tolerance tests

After six hours of fasting, mice were challenged with an intraperitoneal injection of 2 g/kg glucose solution. Blood glucose levels were measured before, and 15, 30, 60, 90, and 120 min after the injection.

### 2.5 Insulin autoantibodies

Serum was obtained from heparinized blood taken by submandibular bleeding and the insulin autoantibody (IAA) titer was subsequently measured using luciferase immunoprecipitation systems (LIPS) assay as described (15).

### 2.6 Multi-color flow cytometry

For the time-course flow cytometry experiments, female and male NOD mice from 2 to 16 weeks of age and new onset diabetic mice were randomly sampled over time and peripheral blood, pancreatic draining lymph nodes (PLN), and pancreas were collected and processed into single cell suspensions prior to staining. Pancreas was minced into small pieces with blades, followed by enzymatic digestion with collagenase at 37°C and purification with 40% Percoll. Peripheral blood was collected in heparin and red blood cells were lysed with NH_4_Cl at 37°C. PLN were crushed through a 70 µM cell strainer. Single cell suspensions were stained with the following antibodies: CD44 (IM7, 11-0441-81, eBioscience, Thermo Fisher Scientific, Waltham, MA), Ly6G (1A8, 551461, BD Pharmigen – BD Biosciences, Franklin Lakes, NJ), CD11b (M1/70, 550993, BD Pharmigen), CD11c (N418, 25-0114-82, BD Pharmigen), CD62L (MEL-14, 17-0621-83, BD Pharmigen), CD4 (GK1.5, 560181, BD Pharmigen), and CD8a (53-6.7, 48-0081-82, eBioscience, Thermo Fisher Scientific). Zombie Aqua™ (Biolegend, San Diego, CA) was used as viability marker. Prior to acquisition, CountBright™ Absolute Counting Beads (C36950, Thermo Fisher Scientific) were added to the samples for quantitation. Cells were acquired with BD Canto AIG and analyzed with FCS 7 Express software (*De Novo*, Pasadena, CA). All analyses were performed on viable singlets as outlined in the gating strategy (**Figure S1**).

### 2.7 Single cell RNA sequencing of peripheral blood and pancreas infiltrating immune cells

#### 2.7.1 CD45^+^ sorting, library preparation, CITE-sequencing

For CITE-sequencing (16), single cell suspensions from peripheral blood and pancreas of four male NOD mice with new-onset disease were prepared according to the procedures outlined above. Cells were labeled using the Biolegend cell hashing protocol (TotalSeq™-A Antibodies and Cell Hashing). Briefly, cells were incubated for 10 min with Fc block, and for 30 min at 4°C with 190 barcoded TotalSeq™-A antibodies (Biolegend) and CD45 antibody (clone 30-F11, Thermo Fisher Scientific). Live CD45^+^ cells were sorted with BD FACS Aria™ II cell sorter (BD Biosciences). After sorting, cells were resuspended in 0.04% BSA in PBS at 1,000 cells/μL and loaded on a GemCode Single-Cell Instrument (10X Genomics, Pleasanton, CA; target recovery of 10,000 cells). Single cell RNA sequencing libraries were prepared with GemCode Single-Cell 3′ V3.1 Gel Bead and Library Kit (10X Genomics) according to the manufacturer’s protocol. The cDNA amplification mix was modified to include ADT PCR additive primer (5’-CCTTGGCACCCGAGAATT*C*C-3’ where * indicates a phosphonothioate bond) and HTO PCR additive primer (5’-GTGACTGGAGTTCAGACGTG TGC*T*C-3’). SPRIselect Reagent kit (Beckman Coulter, Brea, CA) was used for size selection of amplified cDNA for the construction of 3’ gene expression and cell surface protein libraries. Libraries were sequenced on an Illumina HiSeq4000 flow cell at VIB Nucleomics Core (Leuven, Belgium).

#### 2.7.2 Single cell clustering, subsetting and trajectory analysis

Raw sequencing reads were demultiplexed using the Cell Ranger pipeline (10X Genomics) to generate gene expression and cell surface protein matrices. These matrices were further processed to create a Seurat object (Seurat v4.3.0) in R (v4.2.2) (17). Cells expressing >200 genes were retained. Subsequent filtering based on the number of features, unique molecular identifiers (UMIs) and percentage of mitochondrial genes, yielded a final object with 38,648 cells, 24,795 features and 190 protein surface markers. mRNA count matrix was normalized with “LogNormalize”, protein surface marker matrix was normalized with “CLR”. Uniform Manifold Approximation and Projection (UMAP) and clustering (FindClusters, Louvain algorithm) was performed. Cell type identities were annotated based on the expression of canonical protein and gene markers (**Figures S2A–C**). T cell subsetting was performed based on Cd4, Cd8a, Cd44, and Sell gene expression levels (**Figure S3**). Trajectory analysis and pseudotime ordering was performed with the Slingshot package. Clusters annotated based on the expression level of Cd44 and Sell were used as an input to identify trajectories.

#### 2.7.3 Differential gene expression analysis

Differential expressed genes (DEGs) were identified using “FindAllMarkers” and “FindMarkers” from the Seurat software package. DEGs were defined by Wilcoxon Rank Sum Test with default LogFC threshold of 0.25 only testing genes that show a minimum of 25% difference between the groups. P-value adjustment was performed using Bonferroni correction. Volcano plots depict DEGs with a LogFC >0.5 and adjusted P-values <0.001.

### 2.8 Statistics

Statistical analyses were performed using GraphPad Prism software (v9.0)(GraphPad Software, LLC., San Diego, CA). Significant differences were determined by Kruskal-Wallis or Mann–Whitney U test. P-values ≤ 0.05 were considered significant (* ≤ 0.05, ** ≤ 0.01, *** ≤ 0.001, **** ≤ 0.0001). Violin plots represent the data as the median with interquartile ranges. Outliers were removed using the ROUT (Q=1%) method.

## 3 Results

### 3.1 Murine type 1 diabetes develops over distinct stages characterized by progressive immune infiltration and loss of beta cell function

**Figure 1:**
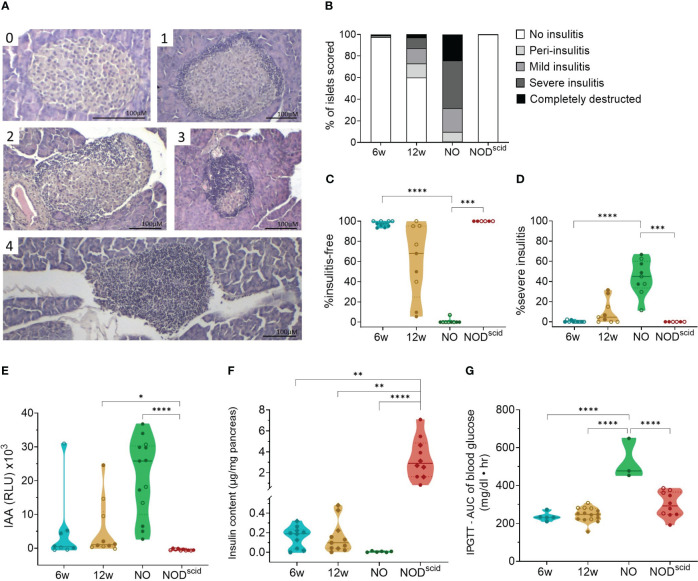
Progressive immune infiltration and loss of beta cell function over the course of disease. Islet immune infiltration and beta cell function was assessed in 6-week-old (6w), 12-week-old (12w), and new-onset (NO) diabetic female (filled circles) and male (empty circles) NOD mice. 12-week-old NOD/scid mice (NOD^scid^) were used as diabetes-resistant congenic control. **(A)** Representative images of H&E-stained islets with the following scoring: (0) no insulitis, (1) peri-insulitis, (2) mild insulitis (<50% of the area), (3) severe insulitis (>50% of the area), and, completely destructed (4). **(B)** Percentage of scored islets with defined degree of insulitis. **(C)** Percentage of insulitis-free and **(D)** of severely infiltrated islets. **(E)** Islets autoantibodies (IAA) titers in the circulation measured by LIPS. **(F)** Insulin content per milligram of pancreas measured by ELISA. **(G)** Area under the curve (AUC) of blood glucose levels upon intraperitoneal glucose tolerance testing (IPGTT). *P ≤ 0.05; **P ≤ 0.01; ***P ≤ 0.001; ****P ≤ 0.0001.

At the outset, we assessed islet immune infiltration and beta cell function by histology and glucose tolerance tests, respectively, in the present NOD cohort. We evaluated three time-points, reflecting the major disease stages: 6 weeks of age, 12 weeks of age, and disease onset. Sex-matched lymphocyte-deficient NOD/scid mice of 12 weeks of age were used as diabetes-resistant congenic control. Islet immune infiltration in both female and male NOD mice significantly progressed during disease development (**Figures 1A, B**). At 6 weeks of age, most of the islets were still insulitis-free (**Figures 1B, C**), whereas at 12 weeks of age, intra-islet immune infiltration increased (**Figures 1B, D**). Despite being inbred, a large interindividual variability in both the proportion of insulitis-free islets (between 5 and 100%) and the severity of the insulitic lesion (between 2 and 30% severely infiltrated islets) could be observed in 12-week-old female and male NOD mice (**Figures 1C, D**). Upon disease onset, insulitis-free islets were absent, and most islets were severely infiltrated or fully destroyed (**Figures 1B–D**). Ongoing beta cell autoimmunity during the pre-symptomatic phase was reflected by the appearance of IAA, which reached peak values at disease onset (**Figure 1E**). Pancreas insulin content inversely correlated with the degree of insulitis during disease progression (**Figure 1F**). Even though 6-week-old NOD mice were devoid of insulitis, their insulin content per milligram of pancreas was already significantly lower compared to insulitis-free NOD/scid mice (>10-fold decrease) (**Figure 1F**), yet they still had a normal intraperitoneal glucose tolerance test (**Figure 1G**). At disease onset, the insulin content was nearly zero (>500-fold decrease compared to NOD/scid) (**Figure 1F**), which was reflected by an aberrant glucose tolerance test (**Figure 1G**).

### 3.2 Dendritic cell trafficking to the draining lymph nodes and neutrophil accumulation in the pancreas

**Figure 2:**
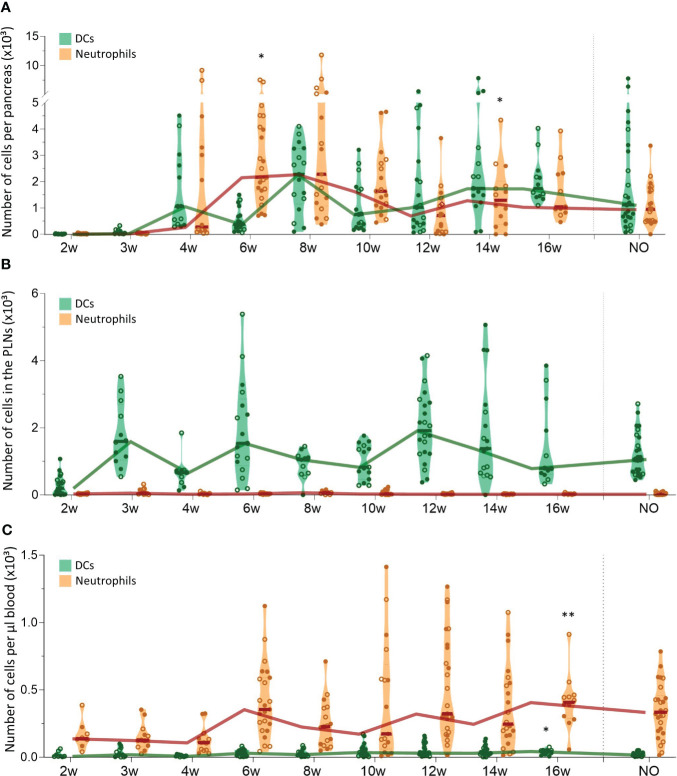
Dendritic cell and neutrophil trafficking throughout disease development. DCs (CD11b^+^CD11c^+^) and neutrophil (CD11b^+^ CD11c^-^Ly6G^+^) numbers in paired **(A)** pancreas, **(B)** pancreatic lymph nodes (PLN), and **(C)** peripheral blood samples from female (filled circles) and male (empty circles) NOD mice of 2 to 16 weeks of age (2w – 16w) and new-onset (NO) diabetic mice were determined with flow cytometry. Data represented as violin plots with median lines visualizing the dynamic profiles over time. Each dot represents an individual mouse. Significant differences between female and male mice are indicated: * p < 0.05, ** p < 0.01.

We evaluated CD11b^+^CD11c^+^ dendritic cell (DC) and CD11b^+^Ly6G^+^ neutrophil trafficking in the pancreas, PLN, and peripheral blood with flow cytometry in both female and male NOD mice between 2 and 16 weeks of age and at disease onset (**Figure 2**). These results revealed that DC numbers fluctuated in waves in the pancreas throughout disease development, with peaks at 4, 8, and 14-16 weeks of age (**Figure 2A**). Notably, DC influxes in the pancreas were consistently preceded by increased DC numbers in the PLN (**Figure 2B**), whereas circulating DC numbers remained constant (**Figure 2C**). These data suggest dynamic DC trafficking between the pancreas and its draining lymph nodes throughout disease development. In contrast, neutrophils accumulated in the pancreas in the early stages of the disease, until 8 weeks of age, where after absolute numbers declined (**Figure 2A**), and they were absent in the PLN (**Figure 2B**). Of note, neutrophil infiltration in the pancreas was not reflected by a concomitant reduction in circulating neutrophil numbers (**Figure 2C**). Taken together, these results demonstrate dynamic DC trafficking between the pancreas and the draining lymph nodes and neutrophil accumulation in the pancreas during early disease stages. Additionally, we observed no major differences in dynamics between female and male NOD mice.

### 3.3 Massive increase in pancreatic T cell numbers during late-stage pre-symptomatic diabetes

**Figure 3:**
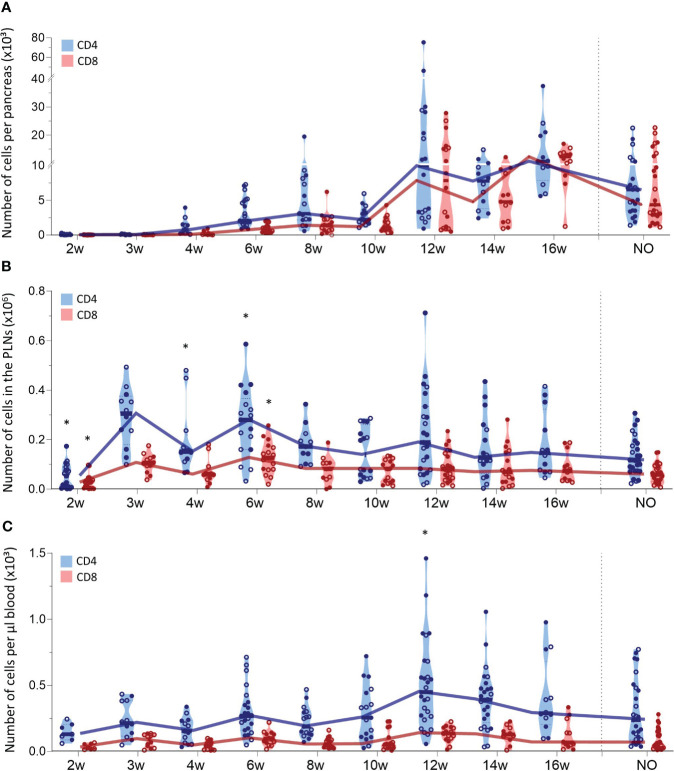
CD4^+^ and CD8^+^ T cell trafficking throughout disease development. CD4^+^ and CD8^+^ T cell numbers in paired **(A)** pancreas, **(B)** pancreatic lymph nodes (PLN), and **(C)** peripheral blood from female (filled circles) and male (empty circles) NOD mice of 2 to 16 weeks of age (2w – 16w) and new-onset (NO) diabetic mice were determined with flow cytometry. Data represented as violin plots with median lines visualizing the dynamic profiles over time. Each dot represents an individual mouse. Significant differences between female and male mice are indicated: * p < 0.05.

We also tracked CD4^+^ and CD8^+^ T cells in the pancreas, PLN, and peripheral blood during T1D development (**Figure 3**). The first CD4^+^ T cells already infiltrated the pancreas at 4 weeks of age and CD4^+^ T cell numbers accumulated over time (**Figure 3A**). CD8^+^ T cells infiltrated the pancreas later than CD4^+^ T cells, as the first CD8^+^ T cells appeared in the pancreas of 6-week-old NOD mice. Similar to CD4^+^ T cells, CD8^+^ T cell numbers accumulated over time. However, CD4^+^ T cells dominated the T cell pool in the pancreas throughout development (**Figure 3A**). Notably, T cell accumulation in the pancreas emerged in two phases. In the early stages of disease development, T cell numbers gradually accumulated, whereas later in the disease course, T cell numbers massively increased in the pancreas. Notably, the time-point in the disease course when this T cell expansion manifested, varied between mice. This T cell expansion was observed from 12 weeks of age onwards. However, whereas for some 12-week-old mice, pancreatic T cell numbers already largely increased, for others, the pancreas was still largely T cell-free (**Figure 3A**). At disease onset, the number of CD4^+^ and CD8^+^ T cells in the pancreas decreased (**Figure 3A**). Comparable to the pancreas, CD4^+^ T cells largely dominated the T cell pool in the PLN (**Figure 3B**). In contrast to T cell accumulation in the pancreas, CD4^+^ and CD8^+^ T cell numbers in the PLNs fluctuated in waves over the course of disease with peaks at 3, 6 and 12 weeks of age, more resembling the dynamics of DC infiltration in the PLNs. In the circulation, the number of CD4^+^ and CD8^+^ T cells per µL of blood remained constant in the early stages of disease development (**Figure 3C**). Concomitant with the increased numbers of T cells in the pancreas, the number of circulating T cells increased at 12 weeks of age. Similar to the dynamics of the innate immune cells, gender had no major impact on T lymphocyte trafficking during disease development. Overall, these results demonstrated continuous CD4^+^ and CD8^+^ T cell trafficking between the pancreas and its draining lymph nodes during disease progression and massive T cell accumulation in late-stage pre-symptomatic diabetes, which was reflected by increased circulating T cell numbers.

### 3.4 A pancreas-specific shift towards T cell effector memory phenotype

**Figure 4:**
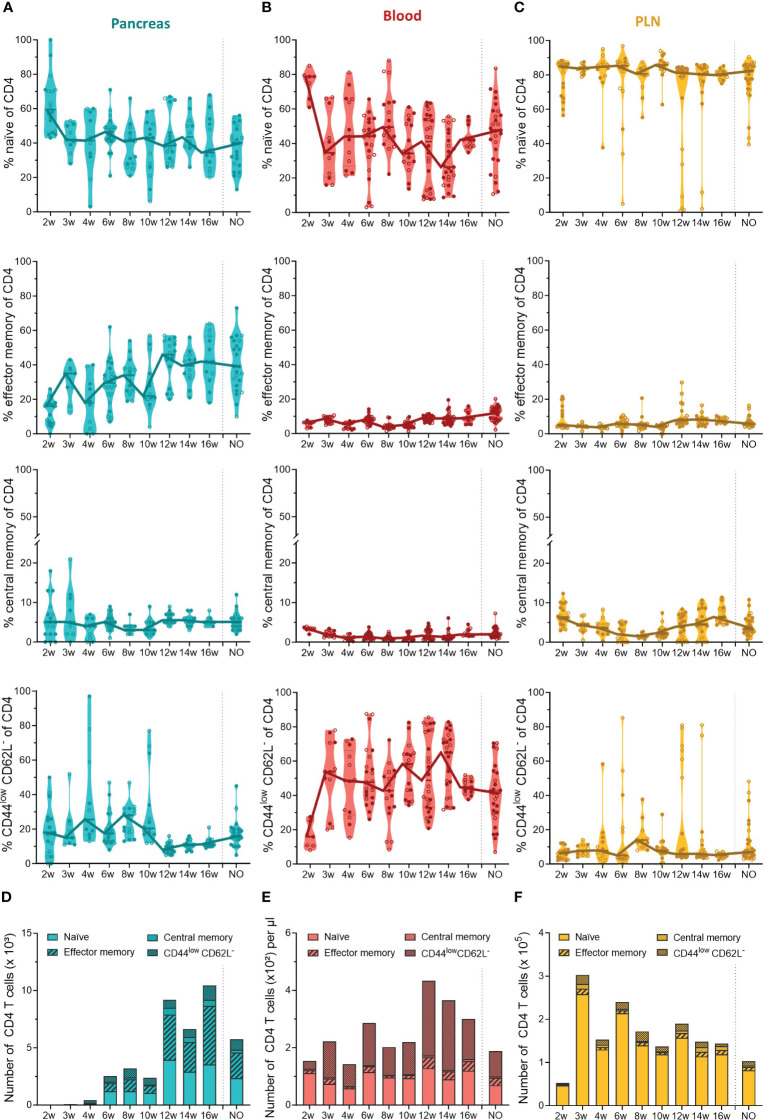
CD4^+^ T cell phenotype in the pancreas and periphery throughout disease development. Percentage of CD4^+^ naïve (CD44^low^ CD62L^high^), effector memory (CD44^high^ CD62L^low^), central memory (CD44^high^ CD62L^high^), and CD44^low^ CD62L^-^ T cells in paired **(A)** pancreas, **(B)** peripheral blood, and **(C)** pancreatic lymph nodes (PLN) from female (filled circles) and male (empty circles) NOD mice of 2 to 16 weeks of age (2w – 16w) and new-onset (NO) diabetic mice were determined with flow cytometry. Data represented as violin plots with median lines visualizing the dynamic phenotypic shifts over time. Each dot represents an individual mouse. Number of CD4^+^ naïve, effector memory, central memory and CD44^low^ CD62L^-^ T cells in the **(D)** pancreas, **(E)** peripheral blood, and **(F)** pancreatic lymph nodes from female and male NOD mice of 2 to 16 weeks of age (2w – 16w) and new-onset (NO) diabetic mice. Data is represented as median stacked bars.

Our data indicated that the first T cells infiltrated the pancreas in early stages of disease development and massively accumulated in the pancreas of late-stage pre-symptomatic mice. In addition to the T cell dynamics, we were also interested in the phenotype of circulating and pancreas infiltrating T cells throughout disease development. Therefore, we determined the T cell activation state based on the expression of CD44 and CD62L. We defined naïve (CD44^low^CD62L^+^), effector memory (CD44^high^CD62L^-^), central memory (CD44^high^CD62L^+^), and CD44^low^CD62L^-^ T cells. These data indicated that a large and relatively constant fraction of the CD4^+^ T cells infiltrating the pancreas throughout disease development were of a naïve phenotype ( ± 40% of CD4^+^ T cells) (**Figure 4A**). Additionally, the frequency of effector memory CD4^+^ T cells in the pancreas increased over the course of disease (**Figure 4A**). As a result, the increase of CD4^+^ T cell numbers observed in the pancreases of 12- to 16-week-old mice (**Figure 3A**) originated from an expansion of both naïve and effector memory T cells (**Figure 4D**). Interestingly, the shift towards a CD4^+^ effector memory phenotype appeared to be specific to the pancreas, as the circulation was largely dominated by naïve and CD44^low^CD62L^-^ CD4^+^ T cells (**Figure 4B**), and the frequency and numbers of circulating effector memory CD4^+^ T cells remained low over time (**Figures 4B, E**). Upon CD4^+^ effector memory expansion in the pancreas (around 12 to 16 weeks of age), the numbers of CD44^low^CD62L^-^ CD4^+^ T cells in the circulation increased (**Figure 4E**). In the PLN, the majority of the CD4^+^ T cell pool was naïve ( ± 90%) (**Figure 4C**). Notably, some mice deviated from this trend and presented a high frequency of CD44^low^CD62L^-^ CD4^+^ T cells instead. In line with a pancreas-specific shift towards an effector phenotype, low frequencies, and numbers of effector memory CD4^+^ T cells were observed over time in the PLN (**Figures 4C, F**).

**Figure 5:**
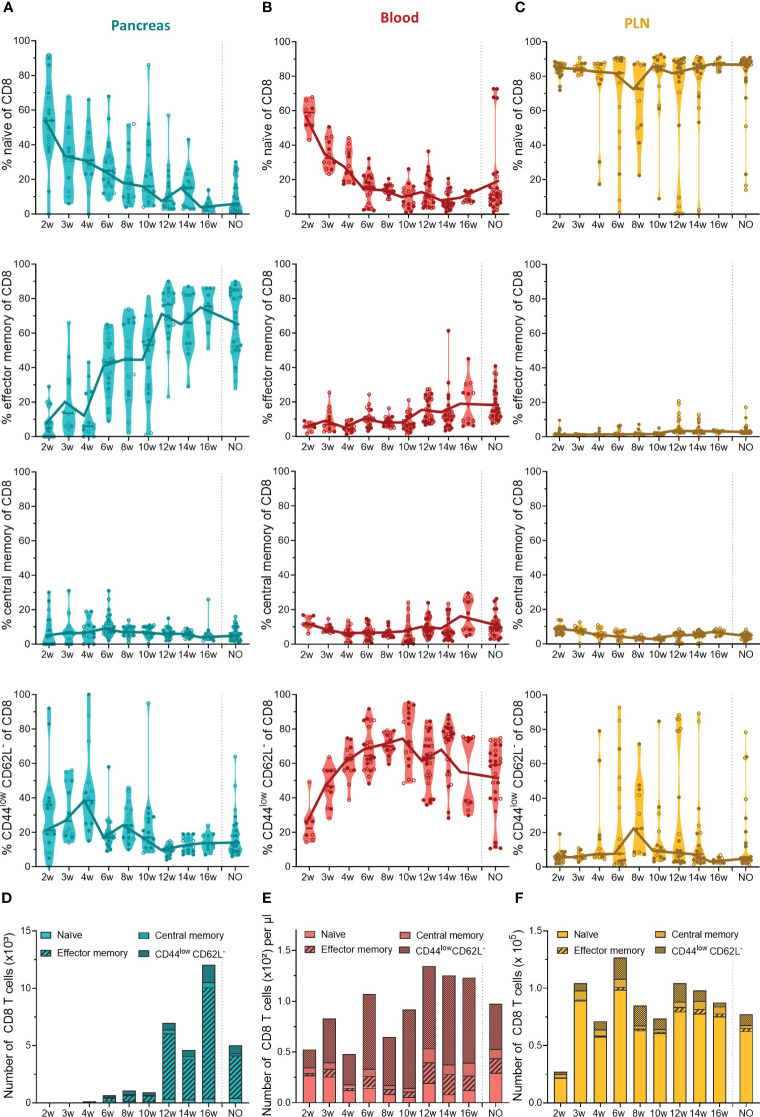
CD8^+^ T cell phenotype in the pancreas and periphery throughout disease development. Percentage of CD8^+^ naïve (CD44^low^ CD62L^high^), effector memory (CD44^high^ CD62L^low^), central memory (CD44^high^ CD62L^high^), and CD44^low^ CD62L^-^ T cells in paired **(A)** pancreas, **(B)** peripheral blood, and **(C)** pancreatic lymph nodes (PLN) from female (filled circles) and male (empty circles) NOD mice of 2 to 16 weeks of age (2w – 16w) and new-onset (NO) diabetic mice were determined with flow cytometry. Data represented as violin plots with median lines visualizing the dynamic phenotypic shifts over time. Each dot represents an individual mouse. Number of CD8^+^ naïve, effector memory, central memory and CD44^low^ CD62L^-^ T cells in the **(D)** pancreas, **(E)** peripheral blood, and **(F)** pancreatic lymph nodes from female and male NOD mice of 2 to 16 weeks of age (2w – 16w) and new-onset (NO) diabetic mice. Data is represented as median stacked bars.

In contrast to the constant pool of naïve CD4^+^ infiltrating T cells, CD8^+^ infiltrating T cells lost their naiveness over time and shifted completely towards an effector memory phenotype (**Figure 5A**). The first CD8^+^ T cells infiltrated the pancreas between 4 and 6 weeks, and already at this early disease stage, around 40% of CD8^+^ T cells were effector memory cells (**Figure 5A**). The phenotypic shift to the CD8^+^ effector memory, combined with the CD8 T cell expansion in the pancreases of 12- to 16-week-old mice (**Figure 3A**), resulted in a massive accumulation of CD8^+^ effector memory T cells during late-stage pre-symptomatic diabetes (**Figure 5D**). Circulating CD8^+^ T cells also lost their naiveness over disease progression (**Figure 5B**). However, identical to CD4^+^ T cells, the shift to effector memory phenotype was confounded to the pancreas, and the circulation was dominated by CD44^low^CD62L^-^ CD8^+^ T cells instead (**Figures 5B, E**). Further supporting a pancreas-specific shift, the frequency, and numbers of circulating effector memory CD8^+^ T cells remained low throughout the disease course. In the PLN, the majority of CD8^+^ T cells were naïve, and similar as observed in the pool of CD4^+^ T cells, some mice deviated from this trend and presented a high frequency of CD44^low^CD62L^-^ CD8^+^ T cells instead (**Figures 5C, F**). Overall, these data demonstrated a shift from naïve to effector memory T cell phenotype over the course of disease development. Interestingly, the circulation was nearly devoid of effector memory T cells, and dominated by CD44^low^CD62L^-^ T cells instead, indicating a pancreas-specific shift to effector memory phenotype and suggesting that an intermediate T cell activation state exists in the circulation.

### 3.5 Transcriptional signature of CD4^+^ and CD8^+^ T cell subsets in pancreas and peripheral blood at disease onset

**Figure 6:**
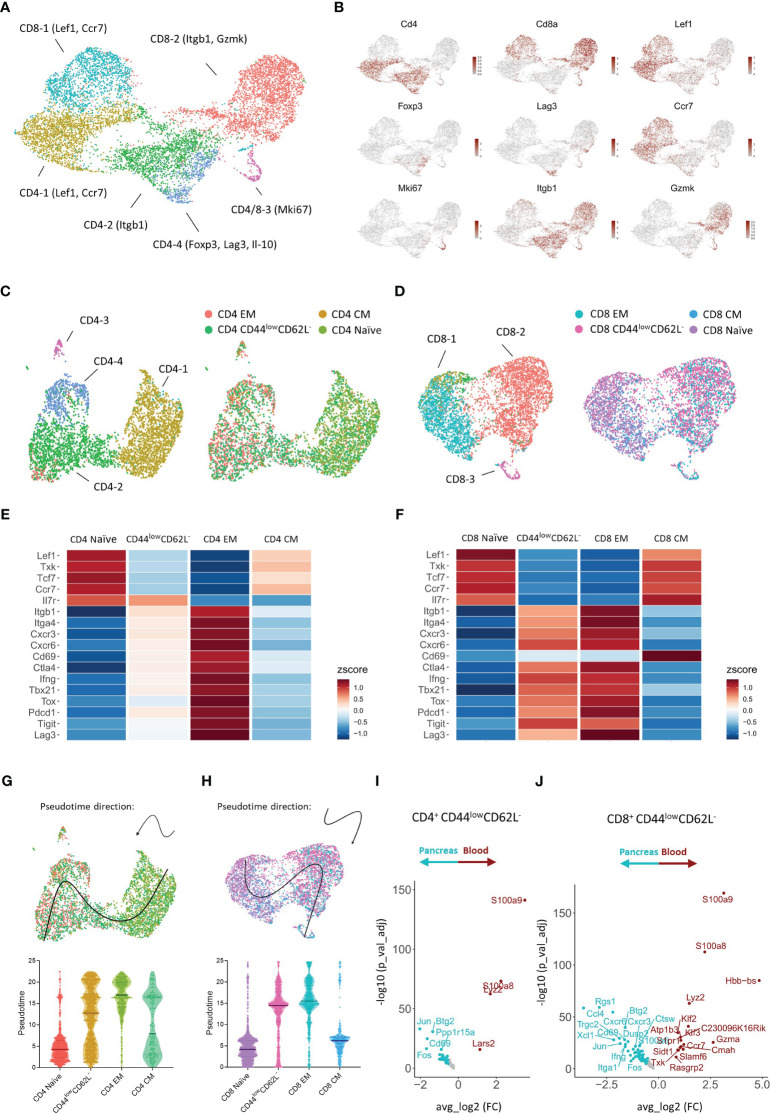
Transcriptional signature of pancreas infiltrating and circulating CD4^+^ and CD8^+^ T cell subsets at disease onset. **(A)** UMAP plot of CD4^+^ and CD8^+^ T cells from peripheral blood and pancreas of new-onset diabetic NOD mice. **(B)** Feature plots depicting gene expression levels of distinct markers driving CD4^+^ and CD8^+^ T cell activation states. **(C, D)** UMAP plot of CD4^+^ **(C)** and CD8^+^ **(D)** T cells grouped by the annotation in A (*left*) and grouped by T cell subset classified based on the expression of Cd44 and Sell (*right*). EM = effector memory (Cd44^+^Sell^-^), CM = central memory (Cd44^+^Sell^+^), naïve (Cd44^-^Sell^+^), and CD44^low^ CD62L^-^ (Cd44^-^Sell^-^). **(E, F)** Heatmap depicting row-based z-scores of scaled gene expression levels for marker genes driving T cell heterogeneity across the distinct CD4^+^ **(E)** and CD8^+^ **(F)** T cell subsets. **(G, H)** Trajectory of CD4^+^ **(G)** and CD8^+^ **(H)** naïve, CD44^low^ CD62L^-^, effector memory (EM), and central memory (CM) T cell subsets projected onto the UMAP plot (*top*). Violin plots depicting the pseudotime of CD4^+^ **(G)** and CD8^+^ **(H)** T cells per subset (*bottom*). Each dot represents a single cell. **(I, J)** Differentially expressed genes of CD4^+^ Cd44^-^Sell^-^ **(I)** and CD8^+^ Cd44^-^Sell^-^ **(J)** T cells between peripheral blood and pancreas.

To our surprise, we did not observe a shift to an effector memory T cell phenotype over time in the circulation, but identified a dominance of T cells expressing low levels of CD44 and CD62L instead. This prompted us to further investigate the biological significance of the different CD4^+^ and CD8^+^ T cell subsets classified based on the expression of CD44 and CD62L. To this extent, we performed CITE-sequencing on CD45^+^ pancreas infiltrating and peripheral blood immune cells from new-onset diabetic NOD mice. We obtained a total of 38,648 cells and 24,795 features and detected the major immune cell populations (**Figure S2A**). Cell type identity was assigned based on the expression of hallmark genes and surface markers (**Figures S2B, C**). Downstream analyses were focused on CD4^+^ and CD8^+^ expressing T cells (**Figure S3A**). A total of 3,924 CD4^+^ and 4,681 CD8^+^ cells pooled from peripheral blood and pancreas were obtained. Expression of hallmark genes revealed different activation states within the CD4^+^ and CD8^+^ T cell pool. For CD4^+^ T cells, these activation states included naïve cells, CD4-1 (Lef1, Ccr7), memory cells, CD4-2 (Itgb1), highly proliferating cells, CD4-3 (Mki67), and regulatory cells, CD4-4, including both classical (Foxp3^+^) and type 1 (Il-10, Lag3) (18). For CD8^+^ T cells, activation marker gene expression indicated naïve cells, CD8-1 (Lef1, Ccr7), effector memory cells, CD8-2 (Itgb1, Gzmk), and highly proliferating cells, CD8-3 (Mki67) (**Figures 6A, B**).

Next, each CD4^+^ and CD8^+^ T cell was classified as either naïve, central memory, effector memory, or CD44^low^ CD62L^-^ based on the expression of Cd44 and Sell (encoding CD62L) (**Figures 6C, D**, **S3B, C**). We then evaluated the expression of distinct markers driving T cell heterogeneity in both CD4^+^ and CD8^+^ T cell subsets (**Figures 6E, F**). CD4^+^ and CD8^+^ naïve T cells expressed higher levels of Lef1, Txk, Tcf7, and the homing receptor Ccr7, which controls chemokine responsiveness to high endothelial venules (HEVs) and mediates homing to lymph nodes (19). CD4^+^ and CD8^+^ effector memory T cells, on the other hand, expressed higher levels of Itgb1 and Itga4, which are typically upregulated upon T cell activation. Similarly, genes implicated in T cell migration (Cxcr3, Cxcr6), T cell activation (Cd69, Ctla4), T cell differentiation (Ifng, Tbx21, Tox, Il7r), and T cell exhaustion (Pdcd1, Tigit, Lag3), were differentially expressed between naïve and effector memory T cells (**Figures 6D, E**). These transcriptional signatures confirm the naïve and effector memory state of CD44^low^CD62L^+^ and CD44^high^CD62L^-^ T cells, respectively. Remarkably, gene expression levels of CD44^low^CD62L^-^ T cells closely resembled those of effector memory T cells, but gene expression levels were not yet completely up- or downregulated compared to effector memory T cells. These results indicated that CD44^low^CD62L^-^ T cells exert a functional state prior to complete differentiation to the effector memory phenotype. In order to better understand the relationship between the CD4^+^ and CD8^+^ naïve, central memory, effector memory and CD44^low^CD62L^-^ T cell subsets, we performed trajectory analysis and pseudotime ordering. These analyses revealed, for both CD4^+^ and CD8^+^ T cells, a trajectory from naïve towards effector memory T cells, and located CD44^low^CD62L^-^ T cells in between these subsets (**Figures 6G, H**). Central memory T cells were positioned between naïve and effector memory T cells. Taken together, these analyses confirm the previous finding that CD44^low^CD62L^-^ T cells exert a functionally intermediate state between naïve and effector memory T cells.

The abundance of this functionally intermediate subset in the circulation and the phenotypic shift to effector memory in the pancreas suggests that CD44^low^CD62L^-^ T cells are further differentiated upon pancreas infiltration. Therefore, we performed DEG analyses between peripheral blood and pancreas. These results indicated distinct transcriptional profiles for CD44^low^CD62L^-^ CD4^+^ and CD8^+^ T cells upon tissue infiltration. For CD4^+^ T cells, genes including Fos, Jun, Cd69 and Btg2 were significantly upregulated in the pancreas (**Figure 6I**). Notably, this gene signature was also found in naïve and effector memory CD4^+^ T cells infiltrating the pancreas (**Figures S4A, B**), as well as in pancreas infiltrating CD8^+^ T cells (**Figures 6J**, **S4C, D**), suggesting that this gene signature is driven by the pancreatic microenvironment. For CD8^+^ T cells, genes including chemokine-encoding genes Ccl4 and Xcl1, chemokine receptor-encoding genes Cxcr3 and Cxcr6 and effector genes Ifng and Itga1 were significantly upregulated in the pancreas (**Figure 6J**). This gene signature, on the other hand, was specific to CD8^+^ CD44^low^CD62L^-^ and effector memory T cells and absent in naïve infiltrating T cells (**Figures S4C, D**), implying that this is an antigen-driven gene response. Taken together, these results demonstrated that circulating CD44^low^CD62L^-^ T cells, which dominated the circulation, exerted a pre-effector-like phenotype. Additionally, our data revealed micro-environment- and antigen-driven transcriptional changes upon pancreas infiltration.

## 4 Discussion

Until date, a thorough comprehension of the intricate immunopathological changes occurring over time within the pancreas is lacking, and impedes progress towards successful therapeutic T1D prevention and/or reversal. Here, we examined the composition of the immune system in pancreatic and peripheral tissues during murine T1D development with multi-parameter flow cytometry. By evaluating absolute numbers of immune cells in multiple disease-relevant organs, and at multiple time-points during the disease course, we provide a unique perspective on the pseudo-spatiotemporal dynamics of DCs, neutrophils, and T lymphocytes during T1D development.

While the role of T cells as direct executors in beta cell loss is well established, less is known on how, when, and where innate immune cells like DCs and neutrophils participate in the destructive process. Neutrophils, as active patrollers of the circulation, have been described in the natural history of T1D over the last years (20, 21). Contradictory evidence exists however on peripheral neutrophil counts and functions like neutrophil extracellular trap (NET) formation during different disease stages (reviewed in (22)), but our group recently demonstrated with high-throughput live-cell imaging that peripheral NETosis was not aberrant in children and adults at various stages of T1D development (23). Still, neutrophils and NET-associated proteins have been shown to accumulate locally in the human pancreas, more specifically in the exocrine part, during the pre-symptomatic stages of T1D and persisted afterwards (5, 24). In the NOD mice as well, neutrophils and NET markers were detected in the pancreas as early as 2 weeks of age (25). Here, we observed that neutrophils indeed accumulated in the pancreas of NOD mice during the early stages of the disease, but disappeared with increasing beta cell loss. However, we cannot make strong conclusions on whether the detected neutrophils resided in the endocrine or exocrine part of the pancreas.

Antigen presenting cells (APCs), including DCs play a central role in initiating an adaptive immune response by activating T cells. We observed waves of DC infiltration in the PLN, starting as early as 3 weeks of age, and found that this dynamic DC infiltration always occurred concomitant with an increase in CD4^+^ and later on CD8^+^ T cell numbers in the PLN. These results suggest waves of T cell activation and expansion upon interaction with cognate antigen-bearing DCs in the draining lymph nodes. Others have also shown that in NOD mice, PLN are crucial for the initial priming of autoreactive T cells beginning at 3 weeks of age (26). However, from 4 weeks of age onwards, the pancreatic islets, and tertiary lymph organs (TLOs) were the sites for priming and clonal expansion of autoreactive T cells, even in the absence of PLN (27). TLOs are organized lymphoid aggregates that arise in perivascular areas in response to chronic inflammation and tissue damage (28). TLOs were found in the pancreas of pre-diabetic NOD mice, and of pre-symptomatic and diabetic human T1D donors, and may represent additional sites of autoreactive effector T cell generation (29–32). We found that DCs infiltrated the NOD pancreas in waves, with the first appearance at 4 weeks of age, a time-point when peri-insulitis transitions to intra-insulitis. With our approach, we are however unable to identify whether DCs were present in the pancreatic islets or in the neighboring exocrine tissue containing TLOs, or in both. Intra-islet DCs were previously shown to expand during disease progression and consisted of distinct subsets, including a CCR7^+^ DC subset, known to be migratory to PLN (18). On the other hand, the T cell area of almost all TLOs contains antigen-loaded DCs (33). Interestingly, depletion of DCs led to the disappearance of existing TLOs (34), suggesting that DCs are imperative for structural organization and maintenance of TLOs, probably as a continuous source of homeostatic and inflammatory chemokines, and of antigen presentation to T cells (reviewed in (33)). Together, our data showed that progressive immune accumulation in the pancreas was characterized by waves of DC infiltration, and DC drainage and T cell expansion in the PLN.

As described by others, we found that T cell infiltration in the pancreas during murine T1D development occurs over two phases (12, 18, 35). From 4 weeks of age onwards, CD4^+^ and CD8^+^ T cells gradually accumulated in small numbers, followed by a marked expansion in late-stage pre-symptomatic mice. By analyzing the changes in the functional state of pancreas infiltrating T cells over the course of disease, we made a few remarkable observations. First, we observed a pancreas-specific shift to an effector memory phenotype. Notably, this pancreas-specific shift was more prominent for cytotoxic CD8^+^ T cells, and resulted in a massive accumulation of CD8^+^ effector memory T cells in the pancreas from 12 weeks of age onwards. We believe that this massive CD8^+^ effector memory expansion in the pancreas represents an important turning point in the disease course, namely the shift from benign towards destructive immune cell infiltration, and marks a critical window of opportunity for therapeutic intervention.

Second, we found that the peripheral blood was dominated by pre-effector-like CD44^low^CD62L^-^ T cells, both CD4^+^ and CD8^+^ T cells. A recent study showed that CD44^low^CD62L^-^CD8^+^ T cells were induced upon tumor inoculation and were linked to anti-tumor activity. They further demonstrated that the CD44^low^CD62L^-^CD8^+^ T cell population was a pre-effector subset, derived from naïve CD44^low^CD62L^high^ T cells, yet incompletely differentiated. Strong TCR signaling, upon anti-CD3/CD28 co-stimulation, differentiated this intermediate subset to CD44^high^CD62L^low^ effector memory T cells (36). Our transcriptional analysis supports the intermediate functional state of both CD4^+^ and CD8^+^ CD44^low^CD62L^-^ subsets in the context of autoimmune diabetes. The CD44^low^CD62L^-^ T cell phenotype was also observed in the PLN during disease progression, strongly suggesting that antigen encounter in the draining lymph nodes may activate naïve CD4^+^ and CD8^+^ T cells towards this intermediate phenotype, followed by migration into the pancreas, and subsequent differentiation into effector T cells. Given its intermediate functional state and predominance in the circulation throughout disease progression, this subset could be a potential novel therapeutic target.

Third, we observed that a large proportion of pancreas infiltrating CD4^+^ T cells were naïve CD44^low^CD62L^high^ T cells, which has been previously observed by others as well (12, 37). This finding indicated that antigenic stimulation was not necessary to traffic and invade the pancreas. Transcriptional analysis confirmed the naïve state of CD44^low^CD62L^high^ expressing T cells. Low expression of *Itga4* and *Itgb1*, the genes encoding for the α (CD49d) and β chain (CD29) of the VLA-4 complex that binds VCAM1 on inflamed islet endothelium, indicates that these cells are poorly equipped to extravasate (38). One possible explanation for the detection of naïve pancreas infiltrating CD4^+^ T cells could be the presence of TLOs. Why these naïve CD4^+^ T cells remain in the pancreas needs further study, but unpublished data from P.L. Bollyky suggest that immature TLOs away from islets act as ‘staging areas’ where naïve T cells assemble before they migrate to the endocrine islets (39). Targeting these local immune niches might be an interesting strategy for disease protection by avoiding the promotion of destructive autoimmunity (27). Yet, caution is needed as these structures have also been linked to more benign disease progression in cancer and infection (reviewed in (28)). Moreover, naïve CD4^+^ T cells could be a pool for local regulatory T cell induction when strong agonistic ligands are provided under sub-immunogenic doses (40).

Insights from our time-course immune mapping sets a framework for the interpretation and design of preclinical and clinical evaluation of immune-modulatory therapeutics. The presence of a therapeutic window of opportunity in late stage pre-symptomatic mice sheds new light on previous inconsistent findings with immunomodulatory drugs. In the past, it was demonstrated that anti-thymocyte globulin treatment showed no effect when initiated at 4 or 8 weeks of age, while treatment of late-stage pre-diabetic (12 weeks of age) and new-onset diabetic mice, mostly around 16 weeks of age, resulted in significant disease prevention and reversal, respectively (41). In a similar fashion, anti-CD3 treatment in early-stage pre-diabetes (4 and 8 weeks of age) showed no effect, whereas treatment at 12 weeks of age and at disease onset induced complete, but transient, protection and remission, respectively (42, 43). We speculate that these immuno-targeted therapies were ineffective when treatment was initiated prior to the turning point of massive cytotoxic CD8^+^ T cell expansion in the pancreas, and that T cell activation is necessary for an effective therapeutic response. This notion reinforces the importance of therapeutic timing for an effective response. In this regard, it has been advocated that treating individuals at-risk early during the disease course might promote favorable outcomes, as more beta cells are still functional. However, too early might carry the risk of even aggravating the disease process and turn benign autoimmunity into destructive insulitis. Additionally, our data revealed significant DC-T cell dynamics over the course of disease having implications for the design of immunotherapies. Given their unique role in the induction and maintenance of self-tolerance, DCs are attractive therapeutic targets for T1D (44). It was previously demonstrated that co-stimulation blockade with CTLA4-Ig was only successful when initiated at 2 to 3 weeks, whereas later administration showed no protection (45). Our data indicated that the first priming events in the PLNs occurred at 3 weeks of age, suggesting that co-stimulation only works prior to the initial priming of autoreactive T cells. Similarly, Abatacept did not prevent progression to stage 2 or 3 in autoantibody positive individuals and the authors noted that the priming of autoreactive T cells had already occurred upon entry, and that the immune response at this stage might not be sensitive to co-stimulation blockade with CTLA4-Ig (46). In addition, we observed waves of DC-T cell activation over the course of disease. Given that disease development in NOD mice is more compact in time, these dynamics could indicate that T1D develops in flares of active disease in human patients. Overall, the abovementioned findings reinforce the value of the NOD mouse as a model to unravel the intricate link between therapeutic mechanism-of-action and efficacy, and disease staging. Nevertheless, due to poor clinical translation, the relevance of the NOD mouse has been questioned (47). We argue that, if used properly, the NOD mouse could be a robust model for preclinical evaluation (48). Disparity in the success of immunotherapies between mice and men could, in part, result from differences between mice and men, one of these being the degree of insulitis. The apparent lack of immune infiltration in some cadaveric samples from individuals with T1D (49) highlights the heterogeneity of the disease, and could, in part, account for the disparity in therapeutic success, given that NOD mice characteristically exhibit immune infiltration in the pancreas and show better response to immunotherapy. Therefore, it could be argued that human subjects should not be included solely based on their seropositivity, but that additional immune-markers should be considered. In this regard, our data also highlighted that it will be important to not only study frequencies and ratios of circulating immune subsets, not limited to their antigen specificities (50), but also their activation states, when making decisions on characteristics, inclusion and timing of disease-modifying therapies.

By analyzing the full pancreas, we were able to capture the dynamics of intra- and extra-islets immune cells, also innate neutrophils, which typically have been out of scope in studies focusing on islets (18). The strength of our study is that we compared these immunodynamics in pancreas, its draining lymph nodes, and the circulation. A limitation to our study was that we lost the spatial context and cell-cell interactions upon sample processing for flow cytometry and single cell RNA sequencing. In conclusion, we identified time- and tissue-dependent changes in immune infiltration dynamics and unraveled different functional states of CD4^+^ and CD8^+^ T cells, that might have important implications for the interpretation and design of preclinical and clinical studies evaluating the potential of disease-modifying therapies and highlight the need for innovative cellular biomarkers of active islet inflammation.

## Data availability statement

The datasets presented in this study can be found in online repositories. The names of the repository/repositories and accession number(s) can be found below: https://www.ncbi.nlm.nih.gov/geo/query/acc.cgi?acc=GSE214031.

## Ethics statement

The animal study was approved by KU Leuven Animal Care and Use Committee. The study was conducted in accordance with the local legislation and institutional requirements.

## Author contributions

YB: Data curation, Formal Analysis, Investigation, Visualization, Writing – original draft, Writing – review & editing. GS: Formal Analysis, Investigation, Writing – review & editing. PJM: Investigation, Writing – review & editing. CG: Conceptualization, Project administration, Supervision, Funding acquisition, Writing – original draft, Writing – review & editing. MV: Data curation, Methodology. CW: Data curation, Writing – review & editing. CM: Supervision, Funding acquisition, Writing – review & editing.

## Supplementary material

Supplementary Figure 1 Representative gating strategy defining T lymphocytes, dendritic cells and neutrophils. Density plots indicating representative gating strategy on the pancreas of a 16 weeks of age female NOD mice. **(A)** Singlet viable cells were selected to gate **(B)** CD4^+^ and CD8^+^ T lymphocyte subsets and **(C)** innate dendritic cells (DCs) and neutrophils. Percentages of parent gate are shown.

Supplementary Figure 2 Major immune cell populations in the pancreas and peripheral blood at disease onset. **(A**) UMAP visualizing major immune cell populations present in the pancreas and circulation of new-onset diabetic mice. **(B)** Feature plots with hallmark genes or surface protein (adt) expression in the major immune cell populations. **(C)** Heatmap showing the top DEGs per immune cell type. Each population was randomly downsized to 200 cells, expect for basophils, which had <200 genes.

Supplementary Figure 3 CD4^+^ and CD8^+^ T cell subsetting based on CD44 and CD62L expression. **(A)** Feature plots (*left*) displaying Cd4 and Cd8a gene expression in the T cell subcluster from **Figure S1A**. FeatureScatter (*right*) visualizing CD4^+^ (red) and CD8^+^ (blue) T cells selected for downstream analysis based on their Cd4 and Cd8a gene expression level. **(B)** CD4^+^ T cells were sub-setted from the T cell sub-clustering shown in . Feature plots (*left*) displaying Sell and Cd44 gene expression in CD4^+^ T cells. FeatureScatter (*right*) visualizing CD4^+^ T cell subsets defined based on their Cd44 and Sell gene expression level. **(C)** CD8^+^ T cells were sub-setted from the T cell sub-clustering shown in . Feature plots (*left*) displaying Sell and Cd44 gene expression in CD8^+^ T cells. FeatureScatter (*right*) visualizing CD8^+^ T cell subsets defined based on their Cd44 and Sell gene expression level.

Supplementary Figure 4 Differential gene expression between pancreas infiltrating and circulating T cell subsets. Volcano plot depicting DEGs between peripheral blood and pancreas of new-onset diabetic NOD mice for **(A)** CD4^+^ naïve, **(B)** CD4^+^ effector memory (EM), **(C)** CD8^+^ naïve, and **(D)** CD8^+^ effector memory (EM) T cells.

## References

[1] Insel RA Dunne JL Atkinson MA Chiang JL Dabelea D Gottlieb PA. Staging presymptomatic type 1 diabetes: a scientific statement of JDRF, the Endocrine Society, and the American Diabetes Association. Diabetes Care (2015) 38(10):1964–74. doi: 10.2337/dc15-1419 PMC5321245 26404926

[2] Mallone R Eizirik DL. Presumption of innocence for beta cells: why are they vulnerable autoimmune targets in type 1 diabetes? Diabetologia (2020) 63(10):1999–2006. doi: 10.1007/s00125-020-05176-7 32894310

[3] Willcox A Richardson SJ Bone AJ Foulis AK Morgan NG. Analysis of islet inflammation in human type 1 diabetes. Clin Exp Immunol (2009) 155(2):173–81. doi: 10.1111/j.1365-2249.2008.03860.x PMC2675247 19128359

[4] Leete P Willcox A Krogvold L Dahl-Jorgensen K Foulis AK Richardson SJ. Differential Insulitic Profiles Determine the Extent of beta-Cell Destruction and the Age at Onset of Type 1 Diabetes. Diabetes (2016) 65(5):1362–9. doi: 10.2337/db15-1615 26858360

[5] Vecchio F Lo Buono N Stabilini A Nigi L Dufort MJ Geyer S. Abnormal neutrophil signature in the blood and pancreas of presymptomatic and symptomatic type 1 diabetes. JCI Insight (2018) 3(18):e122146. doi: 10.1172/jci.insight.122146 30232284 PMC6237216

[6] Damond N Engler S Zanotelli VRT Schapiro D Wasserfall CH Kusmartseva I. A map of human type 1 diabetes progression by imaging mass cytometry. Cell Metab (2019) 29(3):755–68.e5. doi: 10.1016/j.cmet.2018.11.014 30713109 PMC6821395

[7] Wang YJ Traum D Schug J Gao L Liu C Consortium H. Multiplexed *in situ* imaging mass cytometry analysis of the human endocrine pancreas and immune system in type 1 diabetes. Cell Metab (2019) 29(3):769–83.e4. doi: 10.1016/j.cmet.2019.01.003 30713110 PMC6436557

[8] Long SA Thorpe J DeBerg HA Gersuk V Eddy J Harris KM. Partial exhaustion of CD8 T cells and clinical response to teplizumab in new-onset type 1 diabetes. Sci Immunol (2016) 1(5):eaai7793. doi: 10.1126/sciimmunol.aai7793 28664195 PMC5486405

[9] Edner NM Heuts F Thomas N Wang CJ Petersone L Kenefeck R. Follicular helper T cell profiles predict response to costimulation blockade in type 1 diabetes. Nat Immunol (2020) 21(10):1244–55. doi: 10.1038/s41590-020-0744-z PMC7610476 32747817

[10] Suomi T Starskaia I Kalim UU Rasool O Jaakkola MK Gronroos T. Gene expression signature predicts rate of type 1 diabetes progression. EBioMed (2023) 92:104625. doi: 10.1016/j.ebiom.2023.104625 PMC10277927 37224769

[11] Culina S Lalanne AI Afonso G Cerosaletti K Pinto S Sebastiani G. Islet-reactive CD8(+) T cell frequencies in the pancreas, but not in blood, distinguish type 1 diabetic patients from healthy donors. Sci Immunol (2018) 3(20):eaao4013. doi: 10.1126/sciimmunol.aao4013 29429978 PMC5874133

[12] Magnuson AM Thurber GM Kohler RH Weissleder R Mathis D Benoist C. Population dynamics of islet-infiltrating cells in autoimmune diabetes. Proc Natl Acad Sci U S A (2015) 112(5):1511–6. doi: 10.1073/pnas.1423769112 PMC4321317 25605891

[13] Diana J Simoni Y Furio L Beaudoin L Agerberth B Barrat F. Crosstalk between neutrophils, B-1a cells and plasmacytoid dendritic cells initiates autoimmune diabetes. Nat Med (2013) 19(1):65–73. doi: 10.1038/nm.3042 23242473

[14] Rodriguez-Calvo T Ekwall O Amirian N Zapardiel-Gonzalo J von Herrath MG. Increased immune cell infiltration of the exocrine pancreas: a possible contribution to the pathogenesis of type 1 diabetes. Diabetes (2014) 63(11):3880–90. doi: 10.2337/db14-0549 PMC4207385 24947367

[15] Liberati D Wyatt RC Brigatti C Marzinotto I Ferrari M Bazzigaluppi E. A novel LIPS assay for insulin autoantibodies. Acta Diabetol (2018) 55(3):263–70. doi: 10.1007/s00592-017-1082-y 29305766

[16] Stoeckius M Hafemeister C Stephenson W Houck-Loomis B Chattopadhyay PK Swerdlow H. Simultaneous epitope and transcriptome measurement in single cells. Nat Methods (2017) 14(9):865–8. doi: 10.1038/nmeth.4380 PMC5669064 28759029

[17] Hao Y Hao S Andersen-Nissen E Mauck WM 3rd Zheng S Butler A. Integrated analysis of multimodal single-cell data. Cell (2021) 184(13):3573–87.e29. doi: 10.1016/j.cell.2021.04.048 34062119 PMC8238499

[18] Zakharov PN Hu H Wan X Unanue ER. Single-cell RNA sequencing of murine islets shows high cellular complexity at all stages of autoimmune diabetes. J Exp Med (2020) 217(6):e20192362. doi: 10.1084/jem.20192362 32251514 PMC7971127

[19] Berard M Tough DF. Qualitative differences between naive and memory T cells. Immunol (2002) 106(2):127–38. doi: 10.1046/j.1365-2567.2002.01447.x PMC1782715 12047742

[20] Battaglia M Petrelli A Vecchio F. Neutrophils and type 1 diabetes: current knowledge and suggested future directions. Curr Opin Endocrinol Diabetes Obes (2019) 26(4):201–6. doi: 10.1097/MED.0000000000000485 31157631

[21] Petrelli A Popp SK Fukuda R Parish CR Bosi E Simeonovic CJ. The contribution of neutrophils and NETs to the development of type 1 diabetes. Front Immunol (2022) 13:930553. doi: 10.3389/fimmu.2022.930553 35874740 PMC9299437

[22] Bissenova S Ellis D Mathieu C Gysemans C. Neutrophils in autoimmunity: when the hero becomes the villain. Clin Exp Immunol (2022) 210(2):128–40. doi: 10.1093/cei/uxac093 PMC9750832 36208466

[23] Bissenova S Buitinga M Boesch M Korf H Casteels K Teunkens A. High-throughput analysis of neutrophil extracellular trap levels in subtypes of people with type 1 diabetes. Biol (Basel) (2023) 12(6):882. doi: 10.3390/biology12060882 PMC10294788 37372166

[24] Valle A Giamporcaro GM Scavini M Stabilini A Grogan P Bianconi E. Reduction of circulating neutrophils precedes and accompanies type 1 diabetes. Diabetes (2013) 62(6):2072–7. doi: 10.2337/db12-1345 PMC3661622 23349491

[25] Garciafigueroa Y Phillips BE Engman C Trucco M Giannoukakis N. Neutrophil-associated inflammatory changes in the pre-diabetic pancreas of early-age NOD mice. Front Endocrinol (Lausanne) (2021) 12:565981. doi: 10.3389/fendo.2021.565981 33776903 PMC7988208

[26] Gagnerault MC Luan JJ Lotton C Lepault F. Pancreatic lymph nodes are required for priming of beta cell reactive T cells in NOD mice. J Exp Med (2002) 196(3):369–77. doi: 10.1084/jem.20011353 PMC2193939 12163565

[27] Lee Y Chin RK Christiansen P Sun Y Tumanov AV Wang J. Recruitment and activation of naive T cells in the islets by lymphotoxin beta receptor-dependent tertiary lymphoid structure. Immunity (2006) 25(3):499–509. doi: 10.1016/j.immuni.2006.06.016 16934497

[28] Sato Y Silina K van den Broek M Hirahara K Yanagita M. The roles of tertiary lymphoid structures in chronic diseases. Nat Rev Nephrol (2023) 19(8):525–37. doi: 10.1038/s41581-023-00706-z PMC10092939 37046081

[29] Kendall PL Yu G Woodward EJ Thomas JW. Tertiary lymphoid structures in the pancreas promote selection of B lymphocytes in autoimmune diabetes. J Immunol (2007) 178(9):5643–51. doi: 10.4049/jimmunol.178.9.5643 17442947

[30] Henry RA Kendall PL. CXCL13 blockade disrupts B lymphocyte organization in tertiary lymphoid structures without altering B cell receptor bias or preventing diabetes in nonobese diabetic mice. J Immunol (2010) 185(3):1460–5. doi: 10.4049/jimmunol.0903710 PMC3824617 20574003

[31] Smeets S Staels W Stange G Gillard P De Leu N In't Veld P. Insulitis and lymphoid structures in the islets of Langerhans of a 66-year-old patient with long-standing type 1 diabetes. Virchows Arch (2021) 478(6):1209–14. doi: 10.1007/s00428-020-02915-4 PMC8203531 32833039

[32] Korpos E Kadri N Loismann S Findeisen CR Arfuso F Burke GW 3rd. Identification and characterisation of tertiary lymphoid organs in human type 1 diabetes. Diabetologia (2021) 64(7):1626–41. doi: 10.1007/s00125-021-05453-z PMC8187221 33912981

[33] Neyt K Perros F GeurtsvanKessel CH Hammad H Lambrecht BN. Tertiary lymphoid organs in infection and autoimmunity. Trends Immunol (2012) 33(6):297–305. doi: 10.1016/j.it.2012.04.006 22622061 PMC7106385

[34] GeurtsvanKessel CH Willart MA Bergen IM van Rijt LS Muskens F Elewaut D. Dendritic cells are crucial for maintenance of tertiary lymphoid structures in the lung of influenza virus-infected mice. J Exp Med (2009) 206(11):2339–49. doi: 10.1084/jem.20090410 PMC2768850 19808255

[35] Mohan JF Kohler RH Hill JA Weissleder R Mathis D Benoist C. Imaging the emergence and natural progression of spontaneous autoimmune diabetes. Proc Natl Acad Sci U S A (2017) 114(37):E7776–E85. doi: 10.1073/pnas.1707381114 PMC5604023 28839093

[36] Nakajima Y Chamoto K Oura T Honjo T. Critical role of the CD44(low)CD62L(low) CD8(+) T cell subset in restoring antitumor immunity in aged mice. Proc Natl Acad Sci U S A (2021) 118(23):e2103730118. doi: 10.1073/pnas.2103730118 34088845 PMC8201912

[37] Christoffersson G Chodaczek G Ratliff SS Coppieters K von Herrath MG. Suppression of diabetes by accumulation of non-islet-specific CD8(+) effector T cells in pancreatic islets. Sci Immunol (2018) 3(21):eaam6533. doi: 10.1126/sciimmunol.aam6533 29572238

[38] Calderon B Carrero JA Miller MJ Unanue ER. Entry of diabetogenic T cells into islets induces changes that lead to amplification of the cellular response. Proc Natl Acad Sci U S A (2011) 108(4):1567–72. doi: 10.1073/pnas.1018975108 PMC3029718 21220309

[39] Barlow GL Schürch CM Bhate SS Phillips D Young A Dong S. The extra-islet pancreas supports autoimmunity in human type 1 diabetes. (2023) 17:2023.03.15.23287145. doi: 10.1101/2023.03.15.23287145 PMC11999700 40232951

[40] Martens PJ Ellis D Bruggeman Y Viaene M Laureys J Teyton L. Preventing type 1 diabetes in late-stage pre-diabetic NOD mice with insulin: A central role for alum as adjuvant. Front Endocrinol (Lausanne) (2022) 13:1023264. doi: 10.3389/fendo.2022.1023264 36339431 PMC9630573

[41] Simon G Parker M Ramiya V Wasserfall C Huang Y Bresson D. Murine antithymocyte globulin therapy alters disease progression in NOD mice by a time-dependent induction of immunoregulation. Diabetes (2008) 57(2):405–14. doi: 10.2337/db06-1384 18039815

[42] Chatenoud L Thervet E Primo J Bach JF. Anti-CD3 antibody induces long-term remission of overt autoimmunity in nonobese diabetic mice. Proc Natl Acad Sci U S A (1994) 91(1):123–7. doi: 10.1073/pnas.91.1.123 PMC42898 8278351

[43] Chatenoud L Primo J Bach JF. CD3 antibody-induced dominant self tolerance in overtly diabetic NOD mice. J Immunol (1997) 158(6):2947–54. doi: 10.4049/jimmunol.158.6.2947 9058834

[44] Khan FU Khongorzul P Raki AA Rajasekaran A Gris D Amrani A. Dendritic cells and their immunotherapeutic potential for treating type 1 diabetes. Int J Mol Sci (2022) 23(9):4885. doi: 10.3390/ijms23094885 35563276 PMC9099521

[45] Lenschow DJ Ho SC Sattar H Rhee L Gray G Nabavi N. Differential effects of anti-B7-1 and anti-B7-2 monoclonal antibody treatment on the development of diabetes in the nonobese diabetic mouse. J Exp Med (1995) 181(3):1145–55. doi: 10.1084/jem.181.3.1145 PMC2191918 7532678

[46] Russell WE Bundy BN Anderson MS Cooney LA Gitelman SE Goland RS. Abatacept for delay of type 1 diabetes progression in stage 1 relatives at risk: A randomized, double-masked, controlled trial. Diabetes Care (2023) 46(5):1005–13. doi: 10.2337/dc22-2200 PMC10154649 36920087

[47] Roep BO Atkinson M von Herrath M. Satisfaction (not) guaranteed: re-evaluating the use of animal models of type 1 diabetes. Nat Rev Immunol (2004) 4(12):989–97. doi: 10.1038/nri1502 15573133

[48] Shoda LK Young DL Ramanujan S Whiting CC Atkinson MA Bluestone JA. A comprehensive review of interventions in the NOD mouse and implications for translation. Immunity (2005) 23(2):115–26. doi: 10.1016/j.immuni.2005.08.002 16111631

[49] Campbell-Thompson M Fu A Kaddis JS Wasserfall C Schatz DA Pugliese A. Insulitis and beta-cell mass in the natural history of type 1 diabetes. Diabetes (2016) 65(3):719–31. doi: 10.2337/db15-0779 PMC4764143 26581594

[50] Sharma S Tan X Boyer J Clarke D Costanzo A Abe B. Measuring anti-islet autoimmunity in mouse and human by profiling peripheral blood antigen-specific CD4 T cells. Sci Transl Med (2023) 15(703):eade3614. doi: 10.1126/scitranslmed.ade3614 37406136 PMC10495123

