# Supplementary figures and images for: Footprint of pancreas infiltrating and circulating immune cells throughout type 1 diabetes development
Source: Front Endocrinol (Lausanne). 2023 Nov 10;14:1275316. doi: 10.3389/fendo.2023.1275316 (PMC10667927; doi:10.3389/fendo.2023.1275316)

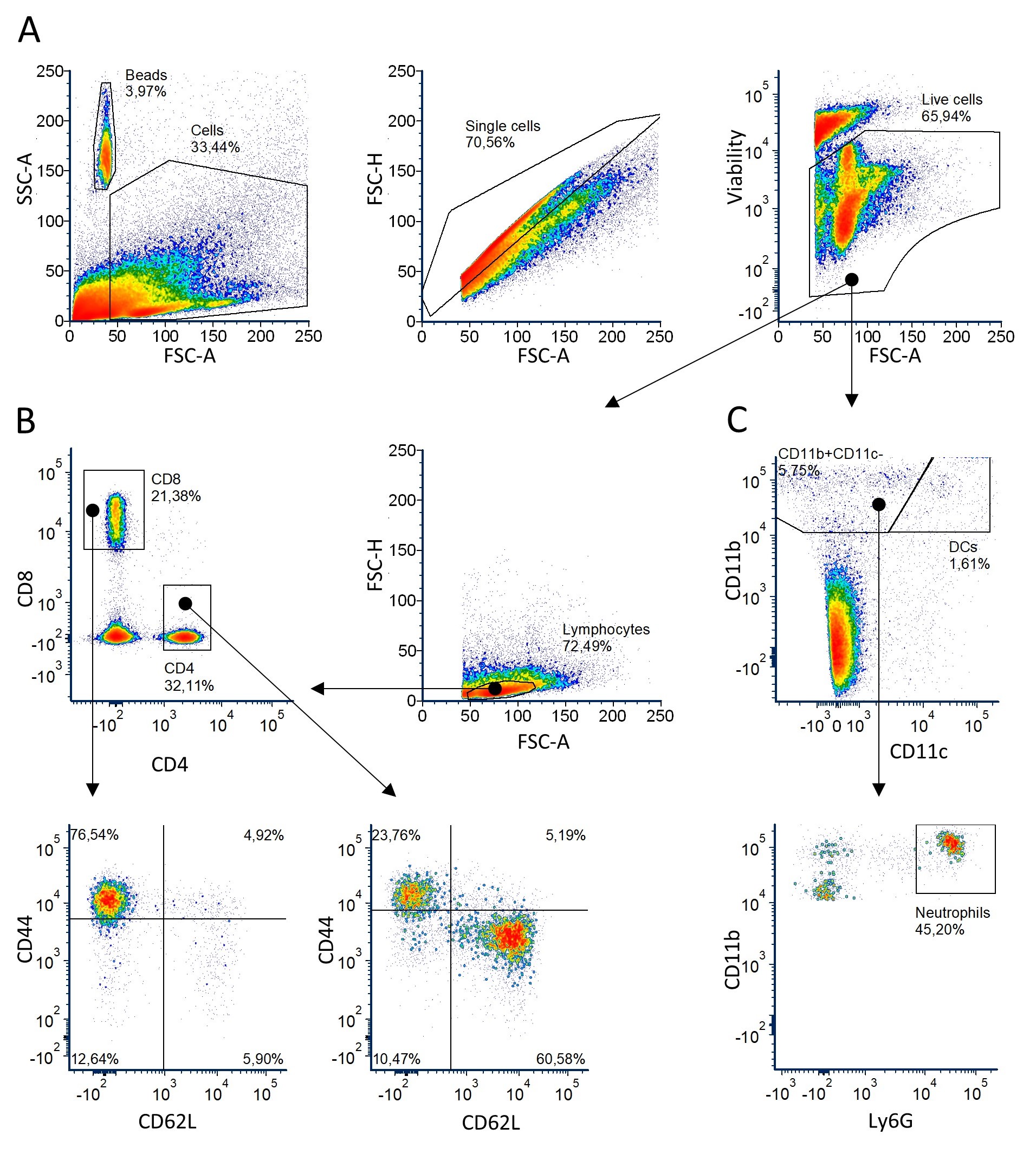

Supplement: Supplementary Figure 1 — Representative gating strategy defining T lymphocytes, dendritic cells and neutrophils. Density plots indicating representative gating strategy on the pancreas of a 16 weeks of age female NOD mice. (A) Singlet viable cells were selected to gate (B) CD4+ and CD8+ T lymphocyte subsets and (C) innate dendritic cells (DCs) and neutrophils. Percentages of parent gate are shown. [file Image_1.jpeg]

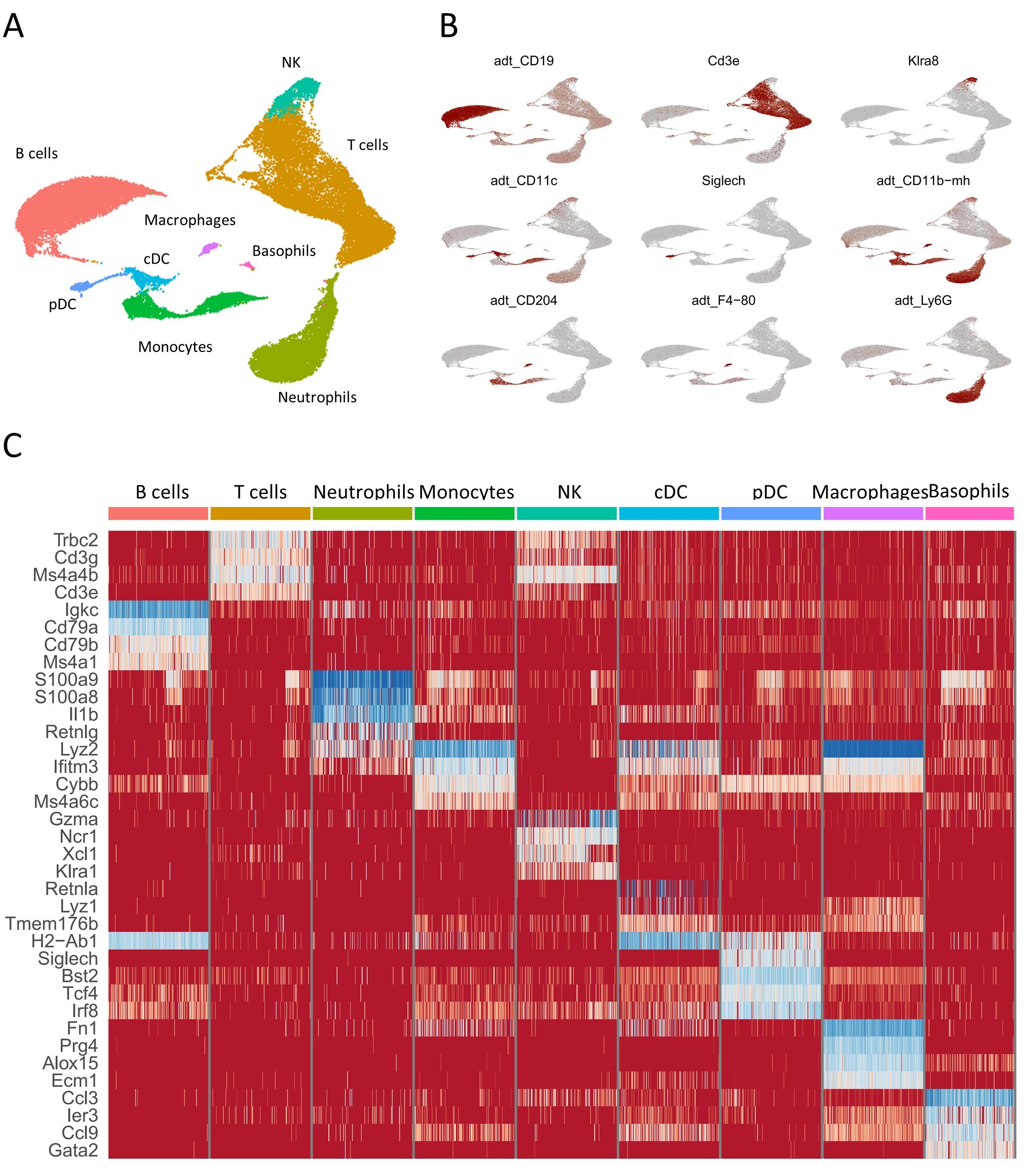

Supplement: Supplementary Figure 2 — Major immune cell populations in the pancreas and peripheral blood at disease onset. (A) UMAP visualizing major immune cell populations present in the pancreas and circulation of new-onset diabetic mice. (B) Feature plots with hallmark genes or surface protein (adt) expression in the major immune cell populations. (C) Heatmap showing the top DEGs per immune cell type. Each population was randomly downsized to 200 cells, expect for basophils, which had <200 genes. [file Image_2.jpeg]

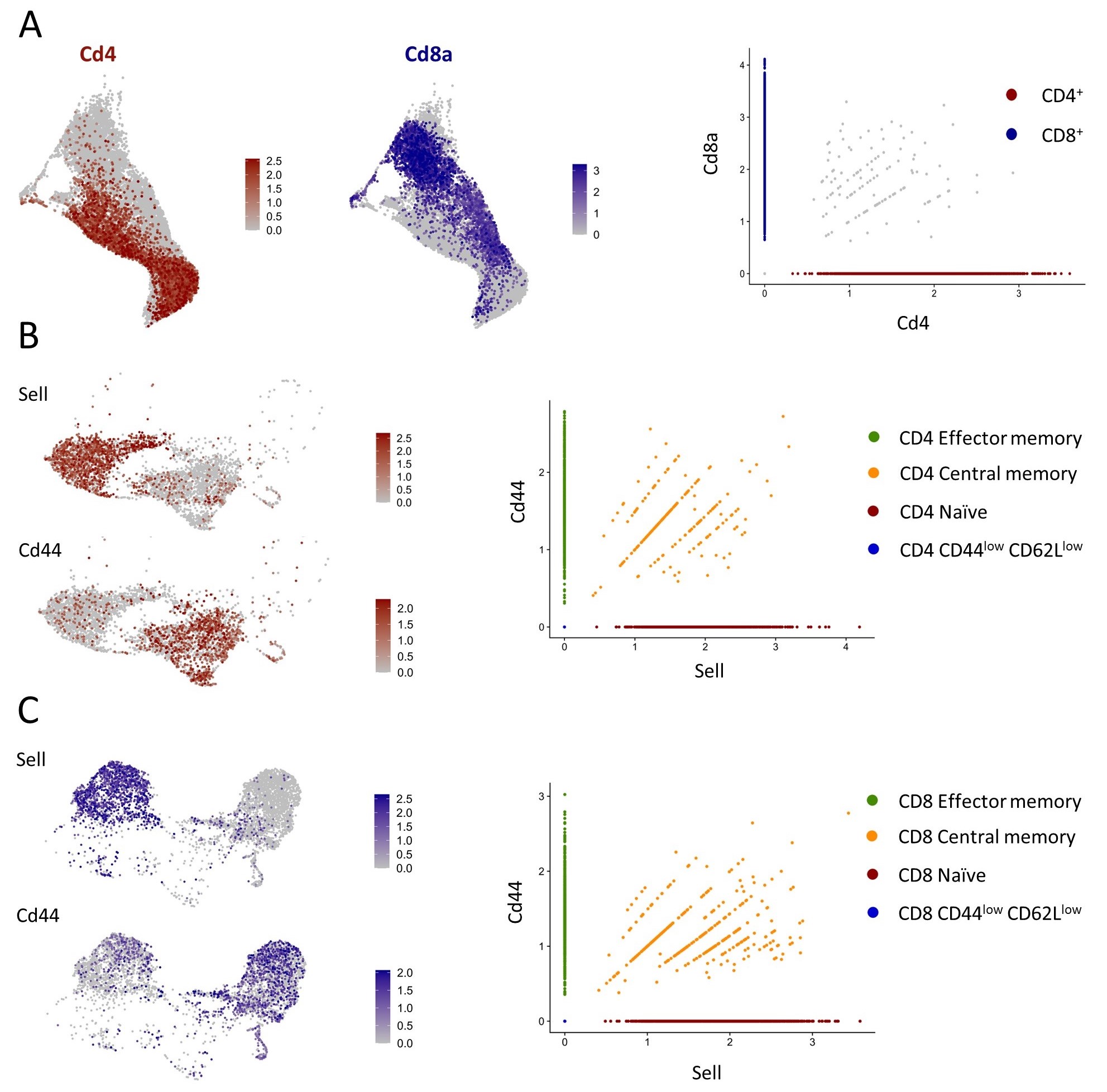

Supplement: Supplementary Figure 3 — CD4+ and CD8+ T cell subsetting based on CD44 and CD62L expression. (A) Feature plots (left) displaying Cd4 and Cd8a gene expression in the T cell subcluster from Figure S1A . FeatureScatter (right) visualizing CD4+ (red) and CD8+ (blue) T cells selected for downstream analysis based on their Cd4 and Cd8a gene expression level. (B) CD4+ T cells were sub-setted from the T cell sub-clustering shown in . Feature plots (left) displaying Sell and Cd44 gene expression in CD4+ T cells. FeatureScatter (right) visualizing CD4+ T cell subsets defined based on their Cd44 and Sell gene expression level. (C) CD8+ T cells were sub-setted from the T cell sub-clustering shown in . Feature plots (left) displaying Sell and Cd44 gene expression in CD8+ T cells. FeatureScatter (right) visualizing CD8+ T cell subsets defined based on their Cd44 and Sell gene expression level. [file Image_3.jpeg]

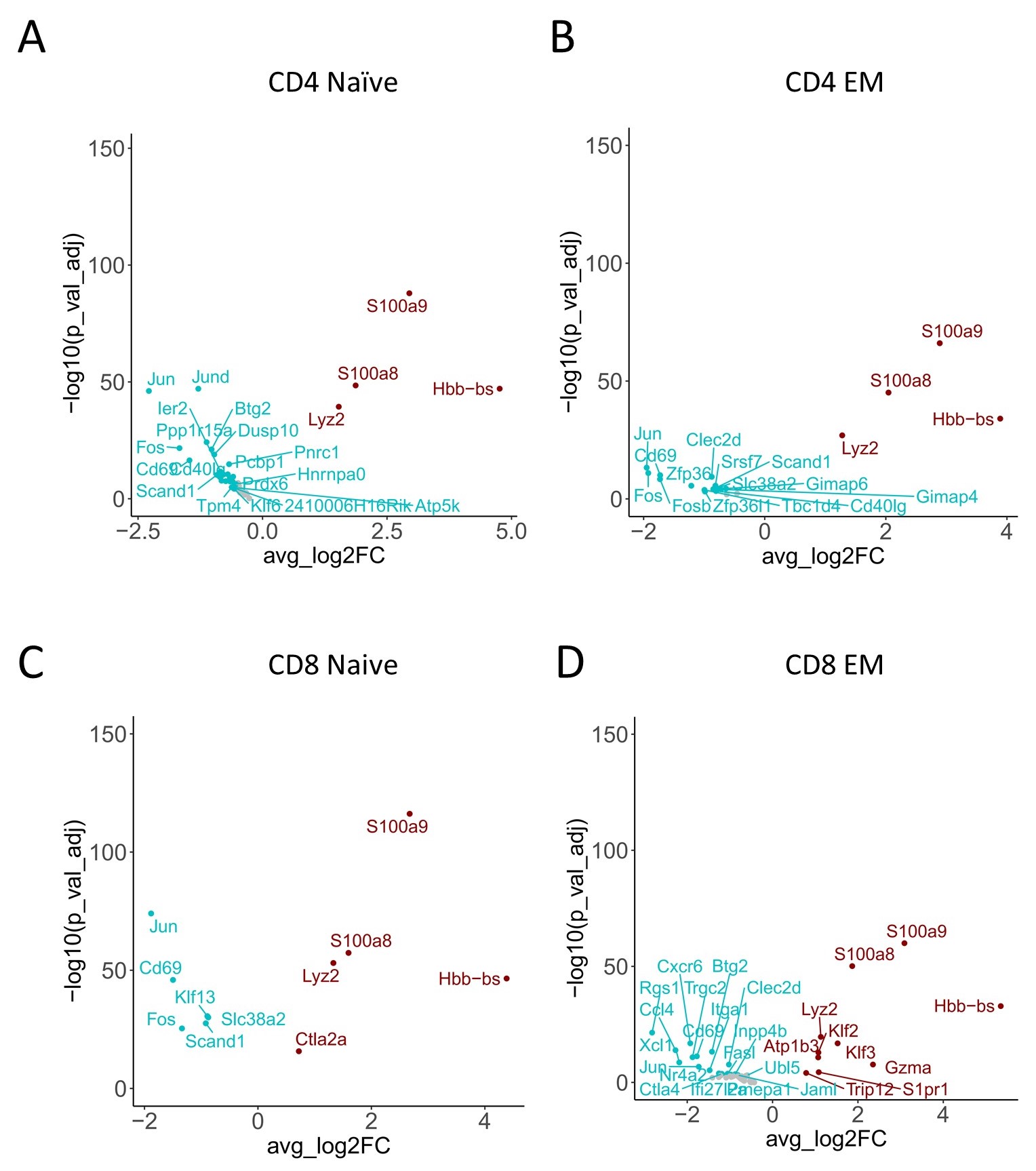

Supplement: Supplementary Figure 4 — Differential gene expression between pancreas infiltrating and circulating T cell subsets. Volcano plot depicting DEGs between peripheral blood and pancreas of new-onset diabetic NOD mice for (A) CD4+ naïve, (B) CD4+ effector memory (EM), (C) CD8+ naïve, and (D) CD8+ effector memory (EM) T cells. [file Image_4.jpeg]
